# Wild-Type Transthyretin Amyloidosis in the Kidneys

**DOI:** 10.1016/j.jaccas.2025.105132

**Published:** 2025-08-20

**Authors:** Tracy Joshi, Tatiana Prokaeva, Deepa M. Gopal, Ashish Verma, Vaishali Sanchorawala, Hui Chen, Eric Burks, Surendra Dasari, Ellen D. McPhail, Andrew Staron

**Affiliations:** a Amyloidosis Center, Boston University Chobanian & Avedisian School of Medicine, Boston, Massachusetts, USA; b Department of Pathology & Laboratory Medicine, Boston University Chobanian & Avedisian School of Medicine, Boston, Massachusetts, USA; c Department of Health Sciences Research, Mayo Clinic, Rochester, Minnesota, USA; d Department of Laboratory of Medicine and Pathology, Mayo Clinic, Rochester, Minnesota, USA

**Keywords:** diagnosis, extracardiac involvement, kidney failure, kidney involvement, wild-type transthyretin amyloidosis

## Abstract

Wild-type transthyretin (ATTRwt) amyloidosis typically presents with restrictive cardiomyopathy. Kidney involvement is exceedingly rare. We report to our knowledge the first antemortem diagnosis of ATTRwt amyloidosis with kidney vasculature deposition in a patient presenting with progressive kidney failure. Notably, technetium-99m pyrophosphate scintigraphy showed no myocardial uptake. This case expands the known spectrum of organ involvement in ATTRwt amyloidosis and underscores the need to consider extracardiac manifestations in its diagnosis.

## Graphical Abstract

**Figure:**
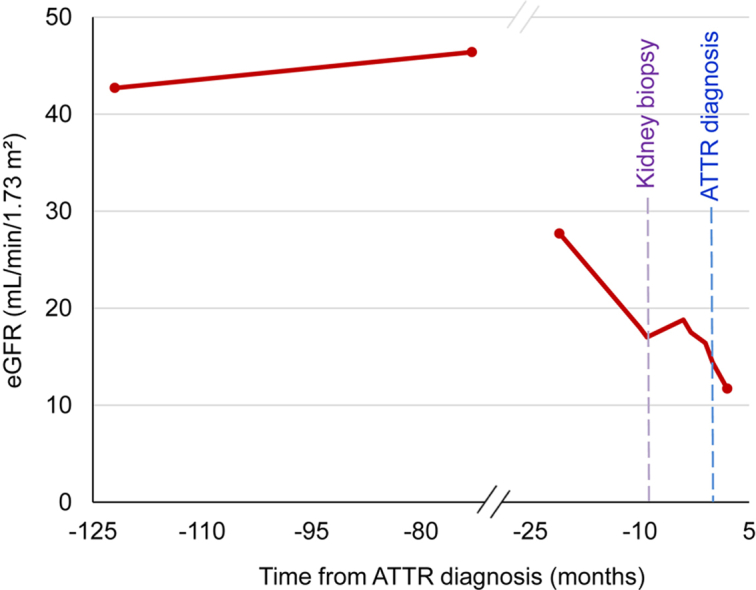

## History of Presentation

**Figure 1:**
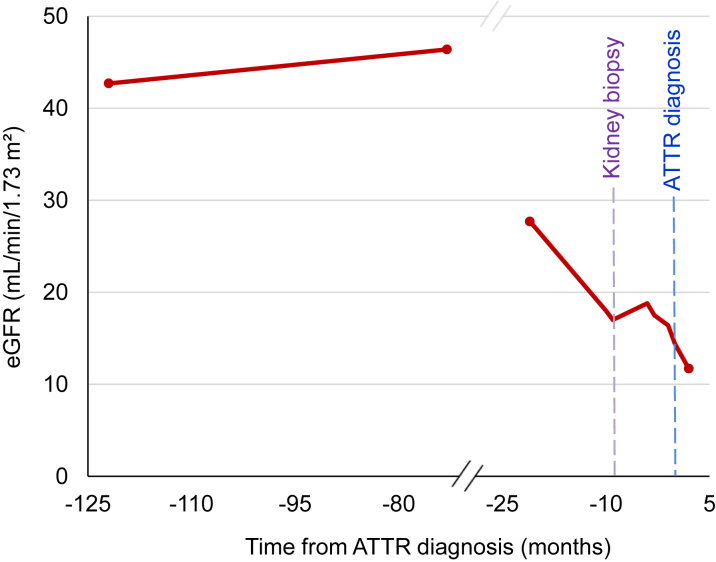
Trajectory of Estimated Glomerular Filtration Rate Relative to Diagnosis The patient maintained stable stage 3a chronic kidney disease for several years, followed by a progressive decline in estimated glomerular filtration rate (eGFR) over the 25 months preceding the diagnosis of transthyretin amyloidosis (ATTR) (time 0).

**Figure 2:**
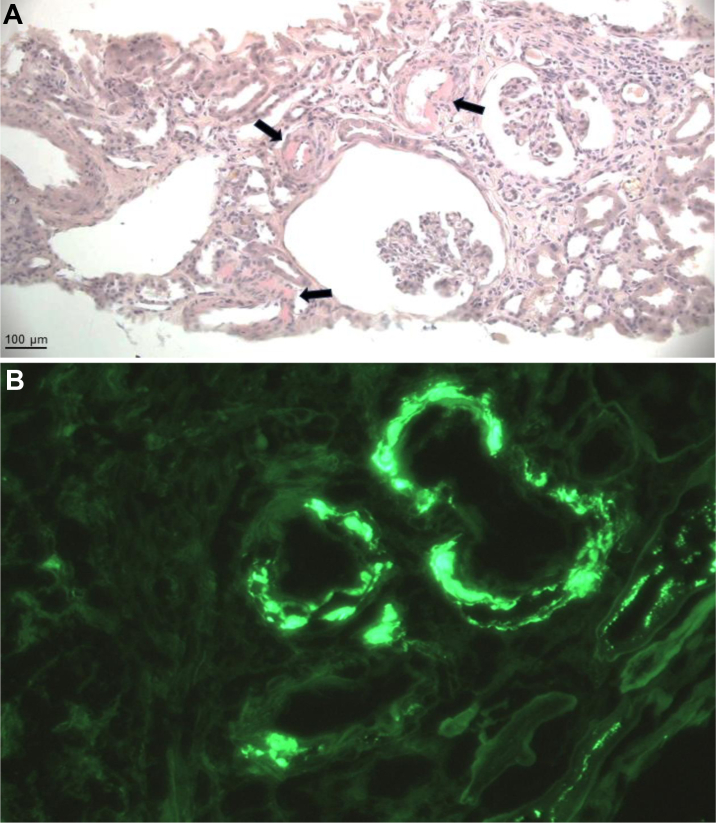
Kidney Biopsy Histology (A) Congo red staining shows perivascular amyloid deposits (arrows) on light microscopy. No amyloid deposits are observed in glomeruli. Original magnification ×3.15. (B) Immunofluorescence staining with kappa light chain antibody. Original magnification ×40.

A 75-year-old man was referred to our center for evaluation of suspected amyloidosis in the context of progressive kidney dysfunction over a 2-year period (Figure 1). Laboratory studies revealed a serum creatinine of 4.11 mg/dL, an estimated glomerular filtration rate of 13 mL/min/1.73 m^2^, and 24-hour urine protein excretion of 649 mg. Congo red staining of kidney biopsy tissue confirmed the presence of amyloid deposits localized to the vascular walls and notably absent from the glomeruli (Figure 2A). Immunofluorescence demonstrated moderate IgG4 and kappa light chain staining within the vascular walls (Figure 2B). Additional histopathologic findings included focal global glomerulosclerosis with ischemic changes, tubular atrophy, interstitial fibrosis, and arteriosclerosis.

**Take-Home Messages**

• ATTRwt amyloidosis can involve extra-cardiac tissues such as the kidneys, typically presenting with vascular-limited deposits and mild proteinuria.
• Accurate diagnosis relies on advanced testing and a multidisciplinary approach to distinguish it from other systemic amyloidoses.

## Past Medical History

The patient had a history of stage 3a chronic kidney disease that remained stable for many years before showing signs of progression (Figure 1). His past medical history was also notable for hypertension, remote pericarditis requiring pericardiectomy, and coronary artery disease complicated by myocardial infarctions and ischemic cardiomyopathy. He had undergone 2 percutaneous coronary interventions, approximately 5 years apart. His orthopedic history included bilateral carpal tunnel syndrome and previous total knee arthroplasty.

## Differential Diagnosis

Based on the immunofluorescence staining pattern observed in the kidney biopsy, both immunoglobulin-derived light chain (AL) and heavy-and-light chain (AHL) amyloidosis were considered in the differential diagnosis. Other amyloid types known to involve the kidneys include leukocyte chemotactic factor 2 (ALECT2), serum amyloid A (AA), and various rare hereditary forms of amyloidosis.^1^

## Investigations

Further work-up revealed no evidence of an underlying plasma cell dyscrasia. Serum and urine immunofixation electrophoresis, along with immunoglobulin enrichment coupled with matrix-assisted laser desorption ionization–time of flight (MALDI-TOF) mass spectrometry, showed no detectable monoclonal protein. Serum free light chain analysis demonstrated kappa and lambda levels of 140.2 mg/L and 70.2 mg/L, respectively, with a kappa-to-lambda ratio of 2.0. These values were within expected reference intervals when adjusted for his kidney function, as estimated by the Chronic Kidney Disease Epidemiology Collaboration equation.^2^ Bone marrow biopsy showed 10% plasma cells without light chain restriction and a normal cytogenetic profile. This modest elevation in plasma cell percentage was more consistent with a reactive polyclonal process than a plasma cell dyscrasia.

**Figure 3:**
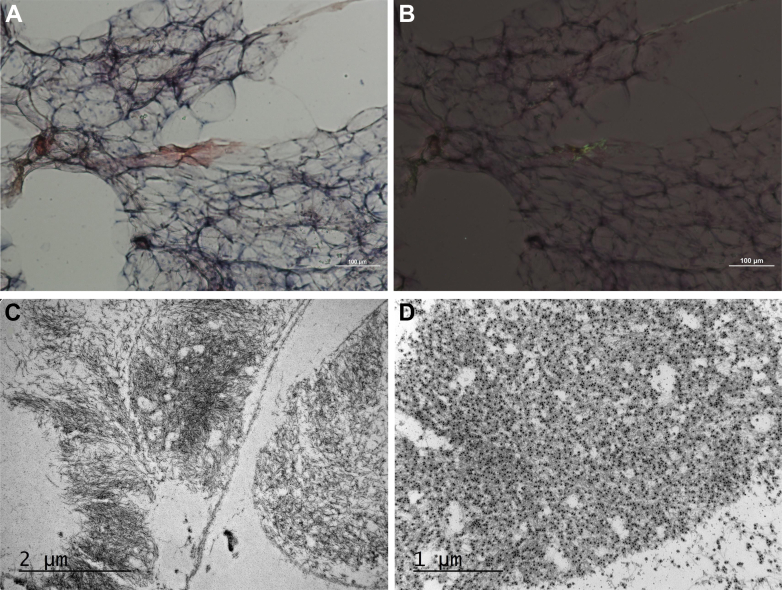
Fat Pad Aspirate Histology (A) Congo red staining shows amyloid deposits on light microscopy. (B) Polarized light microscopy of Congo red–stained tissue demonstrates the characteristic green birefringence of amyloid deposits. Original magnification ×100. (C) Electron micrograph shows extracellular amyloid fibrils with a distinctive haystack-like arrangement. (D) Immunogold labeling shows immunoreactivity with transthyretin-directed antibodies. Original magnification ×15,000.

**Figure 4:**
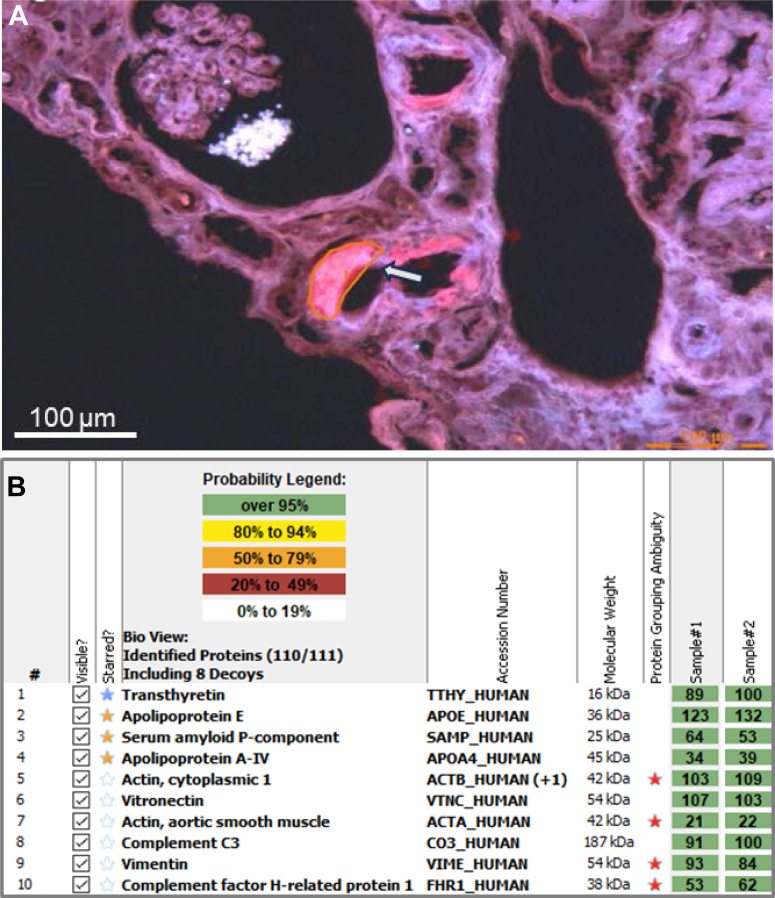
Proteomic Identification of Transthyretin Amyloidosis in Kidney Biopsy Tissue (A) Fluorescence microscopy shows a periarteriolar Congophilic deposit (arrow) microdissected for amyloid fibril typing by LC-MS/MS. Original magnification ×20. (B) Scaffold analysis of Congo red–positive kidney biopsy regions. The blue star indicates the main fibrillar protein (transthyretin), whereas orange stars represent universal amyloid-associated proteins. IgG4 spectra were below background level, and only minimal kappa and lambda light chain spectra were detected with normal ratio, excluding immunoglobulin-derived amyloidosis. No other known amyloidogenic precursor proteins were found. LC-MS/MS = liquid chromatography–tandem mass spectrometry.

An abdominal fat pad aspirate was positive for amyloid by Congo red staining (Figures 3A and 3B). Immunogold electron microscopy of the fat pad identified extracellular, nonbranching amyloid fibrils with immunoreactivity for transthyretin (Figures 3C and 3D). After this result, the kidney biopsy was revisited and analyzed using liquid chromatography–tandem mass spectrometry (LC-MS/MS), which identified the presence of wild-type transthyretin (ATTRwt) peptides in the periarteriolar amyloid deposits and excluded immunoglobulin-derived peptides (Figures 4A and 4B). Genetic testing confirmed no transthyretin gene mutations, supporting the diagnosis of ATTRwt amyloidosis.

**Figure 5:**
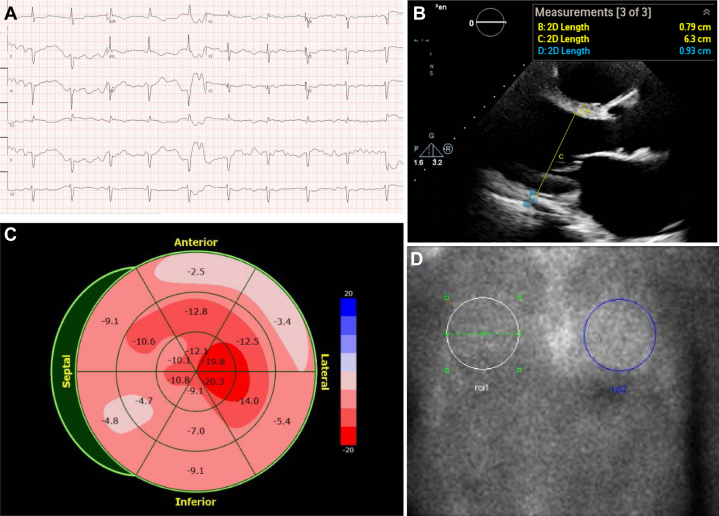
Assessment of Cardiac Involvement (A) Electrocardiogram shows first-degree atrioventricular block, poor R-wave progression, and a pseudoinferior infarct pattern. (B) Transthoracic echocardiography demonstrates an enlarged left ventricle with normal wall thickness. (C) Longitudinal strain bulls-eye plot shows a “cherry-on-top” segmental pattern. Apical segments exhibit preserved strain, whereas the septal and inferior apical segments show reduced function. Severely impaired strain values are observed in the anterior, anterolateral, and inferior segments. Overall, global longitudinal strain is markedly decreased at −9.2%. (D) ^99m^Tc-PYP scan demonstrates Perugini grade 1 myocardial uptake with a heart-to–contralateral lung ratio of 1.19, classified as equivocal for transthyretin cardiac amyloidosis. ^99m^Tc-PYP = technetium-99m pyrophosphate scintigraphy.

Given this diagnosis, cardiac involvement was assessed. The patient had markedly elevated levels of N-terminal pro–B-type natriuretic peptide (37,538 pg/mL), B-type natriuretic peptide (3,097 pg/mL), and high-sensitivity troponin I (156 ng/L). Electrocardiography showed first-degree atrioventricular block (PR interval: 276 ms), normal voltage, poor R-wave progression, and a pseudoinferior infarct pattern in leads II, III, and aVF (Figure 5A). Transthoracic echocardiography demonstrated a moderately enlarged left ventricle (left ventricular end-diastolic diameter indexed to body surface area: 83 mL/m^2^), with normal interventricular septal thickness (8 mm), moderately reduced ejection fraction (33%), septal akinesis, and severe inferior wall hypokinesis (Figure 5B). Global longitudinal strain was reduced at −9.2%, with a “cherry-on-top” apical sparing pattern (Figure 5C). Notably, the apical sparing distribution was discordant with the regional wall motion abnormalities observed. Technetium-99 m pyrophosphate scintigraphy (^99m^Tc-PYP) using nongated planar imaging and single-photon emission computed tomography at 1 hour showed grade 1 myocardial uptake and a heart-to–contralateral lung uptake ratio of 1.19 (Figure 5D), which was considered equivocal for ATTRwt cardiomyopathy.

Our multidisciplinary clinical team concluded that there was no definitive evidence of cardiac involvement by ATTRwt amyloidosis; the patient's cardiac abnormalities were most likely attributable to ischemic heart disease. However, preclinical ATTRwt amyloid cardiomyopathy could not be excluded. Additional diagnostic testing (eg, cardiac magnetic resonance or endomyocardial biopsy) was declined by the patient.

## Management

The patient began treatment with a transthyretin stabilizer, but his condition progressed to end-stage kidney disease, requiring initiation of intermittent hemodialysis. Three months after the diagnosis of ATTRwt amyloidosis, he developed cardiogenic shock secondary to acute coronary syndrome and subsequently passed away.

## Discussion

ATTRwt amyloidosis is primarily recognized as a cardiac disease, with diagnosis and management typically led by cardiologists. However, as a systemic disorder, ATTRwt amyloidosis can involve extracardiac sites—most often tenosynovial tissues, presenting as carpal tunnel syndrome, biceps tendon rupture, and/or lumbar spinal stenosis.^3^ Organ systems such as the lungs, gastrointestinal tract, and genitourinary tract can also be involved.^1^ Diagnosis has traditionally required tissue biopsy, though myocardial scintigraphy using bone-avid tracers has emerged as a reliable noninvasive tool for diagnosing ATTRwt cardiomyopathy in appropriate clinical contexts.^4^

In our patient, ATTRwt amyloid deposits were first identified in kidney biopsy tissue—an unexpected site for this type of amyloidosis. Of the 13 amyloidogenic proteins known to involve the kidneys, those causing AL, ALECT2, and AA amyloidosis are the most prevalent.^1^^,^^5^ Kidney amyloidosis typically affects the glomeruli, leading to proteinuric nephropathy and progressive kidney dysfunction, which can ultimately advance to end-stage kidney disease.^6^^,^^7^

Accurate identification of the amyloid precursor protein is an essential step in the diagnostic work-up of amyloidosis. Immunofluorescence is commonly used for amyloid typing in kidney biopsies; however, it has limitations. Although its sensitivity for detecting immunoglobulin-derived amyloidosis is reported to be 84.6%, false-positive staining for immunoglobulin heavy and/or light chains occurs in up to 12.3% of non-AL amyloidosis kidney biopsies.^5^^,^^8^ In our patient, the low level of proteinuria and absence of a detectable monoclonal protein raised suspicion for misclassification, despite positive immunofluorescence staining for IgG4 and kappa light chains. Amyloid typing by LC-MS/MS subsequently ruled out AL or AHL amyloidosis and confirmed the diagnosis of ATTRwt amyloidosis involving the kidneys. LC-MS/MS is considered the gold standard for amyloid typing, offering a specificity above 98%.^9^

Kidney involvement by ATTR amyloidosis, including both the genetic variant (ATTRv) and wild-type forms of the disease, is exceedingly rare. In a large series comprising 1,890 kidney biopsies analyzed by LC-MS/MS, no cases of ATTR amyloidosis were identified.^1^ Autopsy studies suggest that ATTRwt amyloid may infiltrate kidney tissue and, when present, localizes only to the vasculature.^7^^,^^10^ This is unlike ATTRv amyloid, which has been found to additionally affect the glomeruli and interstitium.^7^^,^^10^ Our patient exhibited vascular-only amyloid deposition with glomerular sparing, a pattern consistent with postmortem observations of ATTRwt amyloidosis and potentially contributing to his kidney failure.

Whether the kidney dysfunction in our patient was directly caused by ATTRwt amyloid deposition in the kidney vasculature remains uncertain. We hypothesize that it was multifactorial, with vascular amyloid, atherosclerosis, and chronic ischemic injury all contributing. Alternatively, the kidney ATTRwt amyloid deposits may have been incidental.

Cardiac involvement in our case was equivocal. The markedly elevated cardiac biomarkers observed may have been influenced by the patient's underlying chronic kidney disease. Echocardiographic strain imaging demonstrated apical sparing, a pattern historically regarded as highly suggestive of cardiac amyloidosis. However, apical sparing can also occur in patients with advanced chronic kidney disease or noninfiltrative heart conditions, such as hypertensive heart disease and ischemic cardiomyopathy. The absence of increased wall thickness was inconsistent with an infiltrative cardiomyopathy in our patient. Furthermore, the ^99m^Tc-PYP scan showed no definitive myocardial radiotracer uptake. Taken together with the clinical context, these findings supported ischemic cardiomyopathy as the more likely etiology. Further evaluation for coronary artery disease was deferred owing to progressive kidney dysfunction.

## Conclusions

This case represents to our knowledge the first antemortem diagnosis of ATTRwt amyloidosis with isolated kidney vascular involvement. It broadens the recognized clinical phenotype of ATTRwt amyloidosis and highlights the importance of considering extracardiac disease manifestations. Precise amyloid fibril typing, preferably by LC-MS/MS, is essential for accurate diagnosis and informs appropriate patient management.

**Visual Summary:** Case Timeline

Timeline	Events
Day 0	Kidney biopsy revealed amyloid deposits with positive immunofluorescence for IgG and kappa light chains, suggesting AHL amyloidosis.
Day 34	Bone marrow biopsy showed <5% plasma cells without light chain restriction. Patient observed.
Day 278	Referred to our center for progression kidney dysfunction. Repeat bone marrow biopsy revealed 10% plasma cells without light chain restriction. Abdominal fat pad aspirate stained positive with Congo red for amyloid deposits.
Day 288	Immunogold electron microscopy of the fat pad identified ATTR amyloidosis.
Day 300	Prior kidney biopsy was typed by LC-MS/MS, confirming a diagnosis of ATTRwt amyloidosis.
Day 317	^99m^Tc-PYP scan was equivocal, showing grade 1 myocardial uptake with a heart-to–contralateral lung ratio of 1.14.
Day 342	Hemodialysis initiated.
Day 371	Hospitalized for acute hypoxic respiratory failure and cardiogenic shock; the patient subsequently passed away.

^99m^Tc-PYP = technetium-99m pyrophosphate scintigraphy; AHL = heavy-and-light chain amyloidosis; ATTR = transthyretin amyloidosis; ATTRwt = wild-type transthyretin amyloidosis; Day 0 = kidney biopsy; LC-MS/MS = liquid chromatography–tandem mass spectrometry.

**Equipment List:** Multimodal Diagnostic Tools

IMAGING	Transthoracic echocardiography system (Philips EPIQ 7) • 3D imaging probe (X5-1 transducer for 3D acquisition) • Strain imaging software (Philips QLAB)
	99mTc-PYP scan system for cardiac amyloidosis evaluation • Radiotracer: ^99m^Tc-PYP (dose per institutional protocol) • 1-hour SPECT/CT imaging protocol with planar views and attenuation correction
HISTOLOGY AND ELECTRON MICROSCOPY	• Congo red stain kit for amyloid detection under bright and polarized light. • Immunoelectron microscopy tools for amyloid typing (antibodies specific to TTR, AA, or AL proteins).
GENETIC ANALYSIS	• DNA was extracted from a portion of the patient's blood sample. • PCR was performed to amplify exons 1-4 of the *TTR* gene. • The PCR products were purified and analyzed using automated nucleotide sequencing in both the forward and reverse directions. • Accuracy and precision of the test, including isoelectric focusing (IEF) and genetic mutation analysis, have been established and verified in compliance with CLIA ’88 standards.
PROTEOMIC ANALYSIS	• Formalin-fixed paraffin-embedded tissue preparation tools. • Laser microdissection system for sample preparation. • Mass spectrometry system (MALDI-TOF and LC-MS/MS) for proteomic analysis.
LABORATORY TESTS AND CLINICAL MONITORING	• Quantitative immunoglobulin assay equipment. • Serum free light chain analysis system. • Immunofixation electrophoresis for blood and urine.

3D = 3-dimensional; ^99m^Tc-PYP = technetium-99m pyrophosphate scintigraphy; AA = serum amyloid A amyloidosis; AL = light-chain amyloidosis; CLIA = Clinical Laboratory Improvement Amendments; LC-MS/MS = liquid chromatography–tandem mass spectrometry; MALDI-TOF = matrix-assisted laser desorption ionization–time of flight; PCR = polymerase chain reaction; SPECT/CT = single-photon emission computed tomography/computed tomography; TTR = transthyretin amyloidosis.

## Funding Support and Author Disclosures

This work was supported by the Windflower Foundation and the Boston University Amyloid Research Fund. These funding sources had no role in the design, writing, or decision to submit this case report. The authors have reported that they have no relationships relevant to the contents of this paper to disclose.
